# Gypenoside XVII Prevents Atherosclerosis by Attenuating Endothelial Apoptosis and Oxidative Stress: Insight into the ERα-Mediated PI3K/Akt Pathway

**DOI:** 10.3390/ijms18020077

**Published:** 2017-02-09

**Authors:** Ke Yang, Haijing Zhang, Yun Luo, Jingyi Zhang, Min Wang, Ping Liao, Li Cao, Peng Guo, Guibo Sun, Xiaobo Sun

**Affiliations:** 1 Beijing Key Laboratory of Innovative Drug Discovery of Traditional Chinese Medicine (Natural Medicine) and Translational Medicine, Institute of Medicinal Plant Development, Peking Union Medical College and Chinese Academy of Medical Sciences, Beijing 100193, China; yangyongyao168@sina.com (K.Y.); hjzforever@126.com (H.Z.); yluo@implad.ac.cn (Y.L.); zhangjingyi707@126.com (J.Z.); mwang@implad.ac.cn (M.W.); lcao@implad.ac.cn (L.C.); pguo@implad.ac.cn (P.G.); 2 Key Laboratory of Bioactive Substances and Resource Utilization of Chinese Herbal Medicine, Ministry of Education, Beijing 100193, China; 3 Zhongguancun Open Laboratory of the Research and Development of Natural Medicine and Health Products, Beijing 100193, China; 4 Key Laboratory of Efficacy Evaluation of Chinese Medicine against Glycolipid Metabolic Disorders, State Administration of Traditional Chinese Medicine, Beijing 100193, China; 5 Department of Pharmacology, Guilin Medical University, Guilin 541000, Guangxi, China; liaoping555@163.com

**Keywords:** gypenoside XVII, estrogen receptors, atherosclerosis, oxidative damage, apoptosis

## Abstract

Phytoestrogens are estrogen-like compounds of plant origin. The pharmacological activities of phytoestrogens are predominantly due to their antioxidant, anti-inflammatory and lipid-lowering properties, which are mediated via the estrogen receptors (ERs): estrogen receptor alpha (ERα) and estrogen receptor beta (ERβ) and possibly G protein-coupled estrogen receptor 1 (GPER). Gypenoside XVII (GP-17) is a phytoestrogen that is widely used to prevent cardiovascular disease, including atherosclerosis, but the mechanism underlying these therapeutic effects is largely unclear. This study aimed to assess the anti-atherogenic effects of GP-17 and its mechanisms in vivo and in vitro. In vivo experiments showed that GP-17 significantly decreased blood lipid levels, increased the expression of antioxidant enzymes and decreased atherosclerotic lesion size in ApoE−/− mice. In vitro experiments showed that GP-17 significantly prevented oxidized low-density lipoprotein (Ox-LDL)-induced endothelial injury. The underlying protective mechanisms of GP-17 were mediated by restoring the normal redox state, up-regulating of the ratio of Bcl-2 to Bax and inhibiting the expression of cleaved caspase-3 in Ox-LDL-induced human umbilical vein endothelial cell (HUVEC) injury. Notably, we found that GP-17 treatment predominantly up-regulated the expression of ERα but not ERβ. However, similar to estrogen, the protective effect of GP-17 could be blocked by the ER antagonist ICI182780 and the phosphatidylinositol 3-kinase (PI3K) antagonist LY294002. Taken together, these results suggest that, due to its antioxidant properties, GP-17 could alleviate atherosclerosis via the ERα-mediated PI3K/Akt pathway.

## 1. Introduction

Cardiovascular disease (CVD) is the major cause of morbidity and mortality worldwide. Atherosclerosis (AS), a major trigger for the development of CVD, is a chronic inflammatory disease of the injured arterial wall, where overproduction of reactive oxygen species (ROS) plays a pivotal role to the development of AS [1]. Although the generation of ROS is the result of multiple factors, an exceptionally important risk factor in the pathogenesis of atherosclerosis is oxidized low-density lipoprotein (Ox-LDL), which contributes to endothelium apoptosis, monocyte adhesion to endothelial cells and other processes [2,3,4]. Numerous critical ROS generated by Ox-LDL-induced endothelial injury activate intracellular signaling, such as activation of the phosphatidylinositol 3-kinase/protein kinase B (PI3K/Akt), disturbance of the balance of Bcl-2 family proteins and the following activation of caspase-9 to initiate a caspase cascade [5,6,7], and activation of heme oxygenase-1 (HO-1). Thus, inhibition of Ox-LDL-mediated ROS production is considered to be a plausible strategy to prevent atherosclerotic diseases.

Notably, clinical and experimental studies have suggested that estrogen plays a major role in the prevention and treatment of AS [8,9], and estrogen has also been shown to inhibit Ox-LDL-induced stimulation of ROS generation and apoptosis in human umbilical vein endothelial cells (HUVECs) [10,11]. Estrogen modulates lipid metabolism, protects against endothelial apoptosis, promotes vasodilatation and repair and counteracts AS [8,9,12]. Decades of research have demonstrated that the biological effects of estrogen are mediated through three distinct receptors: estrogen receptor alpha (ERα), estrogen receptor beta (ERβ), and G protein-coupled estrogen receptor 1 (GPER) [13]. On binding and activating one or more of its three known receptors, estrogen can activate a series of signaling pathways in different cells and tissues. The best-known estrogen signaling involves classical genomic signaling and nongenomic signaling [14]. Previous reports showed that ERα directly interacts with PI3K and regulates its activity in HUVECs [15]. The direct interaction between ERα and PI3K can lead to the downstream signaling cascade of the protein kinase Akt, activating the Nrf2 signaling pathway and inhibiting expression of Bcl-2 family proteins [16,17]. Presently, various types of exogenous estradiol-like agents, such as 17β-estradiol, raloxifene and estrogen-dendrimer conjugates (EDCs), have been used to protect the vascular system [14]. However, the application of estrogen treatment has been hampered by its potential adverse effects, including blood clots, headache, breast pain, endometrial stimulation, breast cancers and various other disorders [18]. Because of these concerns, it is important to investigate other estrogen-like agents that can mechanistically delay or prevent the onset and progression of AS.

**Figure 1:**
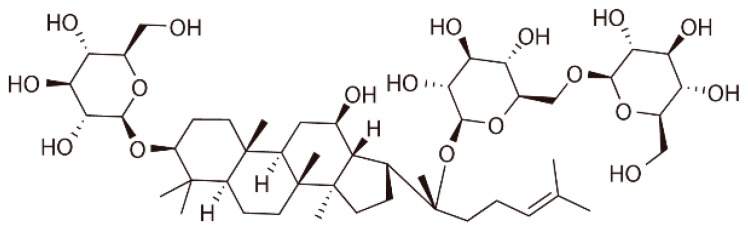
The chemical structure of GP-17.

Phytoestrogens are compounds with structural similarities to estradiol that can bind weakly to estrogen receptors and exert estrogenic properties [19]. They have protective effects against AS in vivo and in vitro [20]. The mechanisms for the anti-atherogenic effects of phytoestrogens may be associated with estrogen receptor-dependent genomic and nongenomic signaling. Gypenoside XVII (GP-17), a novel phytoestrogen belonging to the gypenosides, has a similar structure to that of estradiol (Figure 1). Gypenosides, which are extracted from *Gynostemma pentaphyllum*, have various pharmacological properties and protect against cardiovascular diseases, especially atherosclerosis [21,22,23]. Previously, our team found significant neuroprotective effects of GP-17 against oxidative stress and apoptosis induced by the ERs/PI3K/Akt/HO-1 pathway [16]. However, whether GP-17 has anti-atherogenic effects is poorly understood.

Based on this evidence, we first used the in vivo model of apolipoprotein E-deficient (ApoE−/−) mice to verify the anti-atherogenic effect of GP-17. To fully elucidate the beneficial mechanisms of GP-17, we further investigated a model of Ox-LDL-induced HUVEC injury in vitro and found that the major mechanism of this action relied on the estrogen-like activity of this compound. For the first time, the significant anti-atherogenic effects of GP-17 against endothelial cell apoptosis and oxidative stress were elucidated.

## 2. Results

### 2.1. GP-17 Suppresses Atherosclerotic Development in ApoE−/− Mice

**Figure 2:**
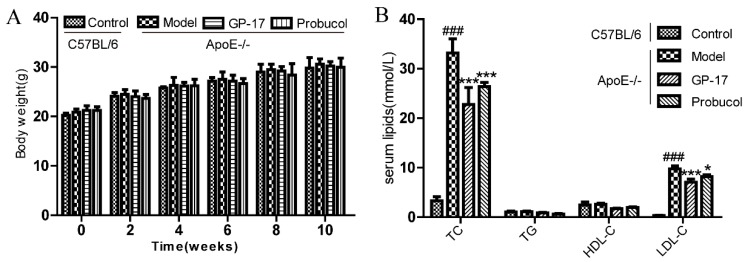
Effects of GP-17 on the body weights and serum lipids at 10 weeks in high fat diet-induced atherosclerotic ApoE−/− mice. (**A**) Changes in body weight after treatment; (**B**) The level of serum lipids. The values are expressed as the mean ± S.E.M. (*n* = 8 for each group). ^###^ *p* < 0.001 vs. Control; * *p* < 0.05; *** *p* < 0.01 vs. Model. TC, total cholesterol; TG, triglyceride; HDL-C, high-density lipoprotein cholesterol; LDC-C, low-density lipoprotein cholesterol.

Body weights were measured as physical measures of hormone bioactivity. Mean body weights were significantly higher in every group compared to that of the control, but there was no significant difference in body weight between the different treatments during the 10-week feeding (Figure 2A). The mouse plasma lipid levels were also measured at the end of 10 weeks of a high-fat diet. Circulating levels of total cholesterol (TC) and low-density lipoprotein cholesterol (LDL-C) were significantly increased in the treated groups of ApoE−/− mice compared with those of the C57BL/6J control group; however, GP-17 and probucol treatment substantially decreased both of these parameters relative to those of the ApoE−/− model group. Additionally, there was no difference in HDL-C or TG between the ApoE−/− mice and C57BL/6J mice (Figure 2B).

**Figure 3:**
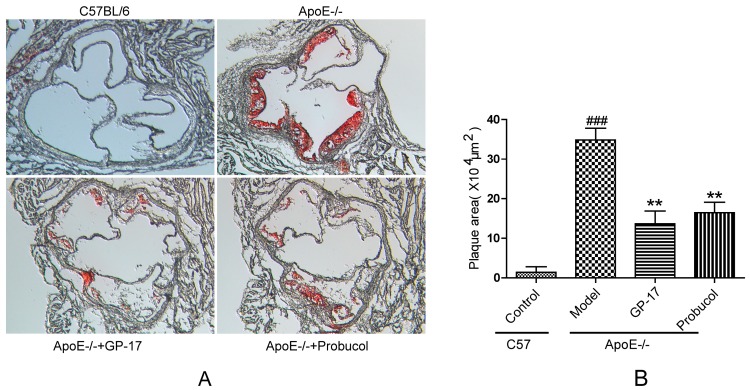
GP-17 effects on lipid deposition in the aortic sinus stained with oil red O. (**A**) Atherosclerotic lesion shown with oil red O staining; (**B**) Quantitative analysis of plaque areas in the aortic sinus by Image-Pro Plus software. Original magnification (**A**): ×200. The values are expressed as the mean ± S.E.M. (*n* = 8 for each group). ^###^ *p* < 0.001 vs. Control; ** *p* < 0.01 vs. Model.

Lipid deposition was used to analyze the effect of oral GP-17 in the aortic root. The results showed that the control group had scarcely any visible lesions stained with oil red O. However, the model group had prominent atherosclerotic lesions compared with those of the control group. Moreover, compared with the model group, GP-17- and probucol-treated ApoE−/− mice had a substantially decreased atherosclerotic lesion area (Figure 3A,B).

### 2.2. GP-17 Mitigates Oxidative Stress in Serum

**Figure 4:**
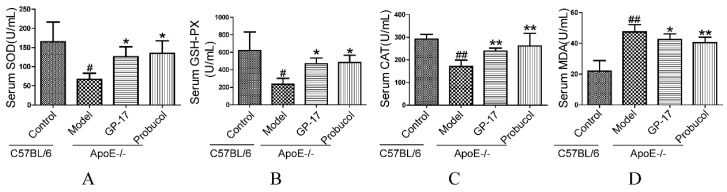
GP-17 increased the levels of SOD, GSH-Px and CAT and decreased MDA content in the serum. (**A**) Serum SOD level; (**B**) Serum GSH-Px level; (**C**) Serum CAT level; (**D**) Serum MDA level. The values are expressed as the mean ± S.E.M. (*n* = 8 for each group). ^#^ *p* < 0.05; ^##^ *p* < 0.01 vs. Control; * *p* < 0.05; ** *p* < 0.01 vs. Model.

Since estrogen can reduce superoxide levels in the serum, the antioxidant effects of GP-17 in the serum were measured. In the GP-17 group, the levels of superoxide dismutase (SOD), glutathione peroxidase (GSH-Px) and catalase (CAT) were significantly higher than those in the model group (Figure 4A–C). However, MDA level, which is a marker of oxidative damage, was decreased in the GP-17-treated group, and there were significant differences between the GP-17 group and model group (Figure 4D).

### 2.3. Protective Effect of GP-17 on Ox-LDL-Induced Cytotoxicity in HUVECs

**Figure 5:**
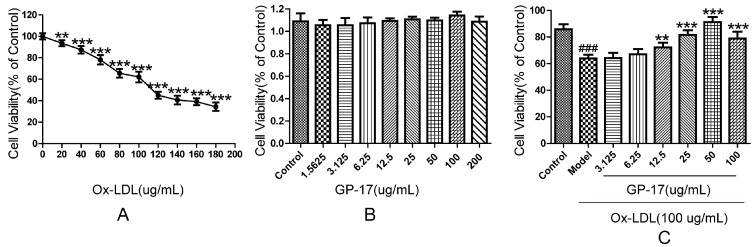
Cytoprotective effects of GP-17 on Ox-LDL-induced cytotoxicity in HUVECs. (**A**) HUVECs were treated with Ox-LDL at different concentrations for 24 h. Cell viability was measured by MTT assays; (**B**) Cell viability of HUVECs incubated with different concentrations of GP-17 for 12 h; (**C**) Incubation with GP-17 significantly lowered Ox-LDL-induced cell injury. Cell viability was measured by MTT assays. The values are expressed as the mean ± S.E.M. from three independent experiments. ^###^ *p* < 0.001 vs. Control; ** *p* < 0.01; *** *p* < 0.001 vs. Model. MTT, 3-(4,5-dimethylthiazol-2yl)-2,5-diphenyltetrazolium bromide.

The ability of GP-17 to prevent Ox-LDL-induced cytotoxicity was detected by cell viability assays. We first examined the cytotoxicity of Ox-LDL in HUVECs. As shown in Figure 5A, Ox-LDL at concentrations of more than 100 μg/mL significantly reduced the viability of HUVECs; thus, 100 μg/mL was selected for subsequent experiments. Second, we examined the cytotoxicity of GP-17 in HUVECs. Figure 5B shows that GP-17 did not demonstrate any cytotoxicity. Finally, we evaluated whether GP-17 could protect HUVECs against Ox-LDL-induced apoptosis. Figure 5C shows that GP-17 dose-dependently mitigated the toxic effect of Ox-LDL on HUVEC viability. The viability of HUVECs was significantly higher than that of other groups at 50 μg/mL GP-17, so 50 μg/mL was selected for subsequent experiments. However, GP-17 counteraction of Ox-LDL-induced cell injury was not time—(Figure S1). Taken together, these data indicated that GP-17 could protect endothelial cells from Ox-LDL-induced cytotoxicity.

### 2.4. GP-17 Inhibits Ox-LDL-Induced Apoptosis in HUVECs

**Figure 6:**
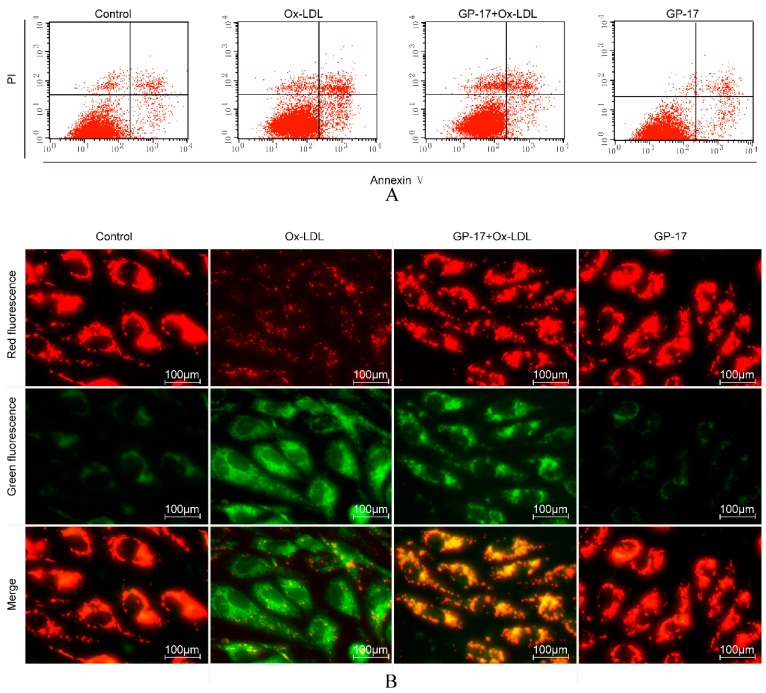

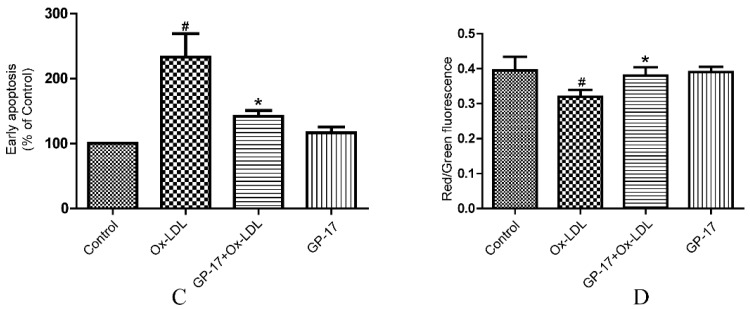
GP-17 protected against Ox-LDL-induced apoptosis in HUVECs. (**A**) Apoptosis in HUVECs was analyzed by flow cytometry; (**B**) ΔΨm was assessed by fluorescence microscopy; (**C**) Quantitative analysis of the percentages of early apoptotic cells; (**D**) Quantitative analysis of ΔΨm by plate reader. The values are expressed as the mean ± S.E.M. from three independent experiments. ^#^ *p* < 0.05 vs. Control; * *p* < 0.05 vs. Ox-LDL. PI, propidium iodide.

The induction of apoptosis in Ox-LDL-induced HUVECs was evaluated using Annexin V-FITC/PI staining assays and JC-1 staining. With Annexin V-FITC/PI staining, the results demonstrated that the number of early apoptotic cells was significantly increased in Ox-LDL-treated HUVECs compared with that of the control group. Incubation with GP-17 (50 μg/mL) significantly reduced the number of early apoptotic cells, while GP-17 alone did not significantly increase apoptosis of HUVECs compared with that of the control group (Figure 6A,C).

Mitochondria also play a key role in the process of cell apoptosis, and the disruption of mitochondrial membrane potential (ΔΨm) is an early event in the apoptotic cascade. To further test the anti-apoptotic effect of GP-17, we examined the protective effect of GP-17 on mitochondrial permeability. We found that the ΔΨm of the mitochondria was depolarized in cells treated with Ox-LDL, as shown by the decrease in red fluorescence and increase in green fluorescence compared with that of the control group, but it was reversed by pretreatment with GP-17 (Figure 6B,D).

### 2.5. GP-17 Reduced Ox-LDL-Induced ROS Generation in HUVECs

**Figure 7:**
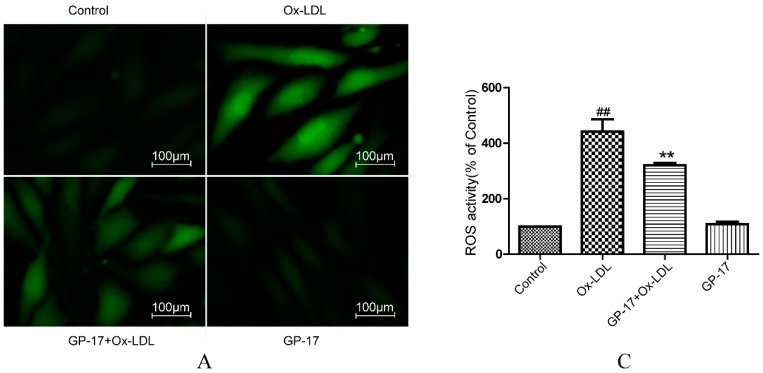

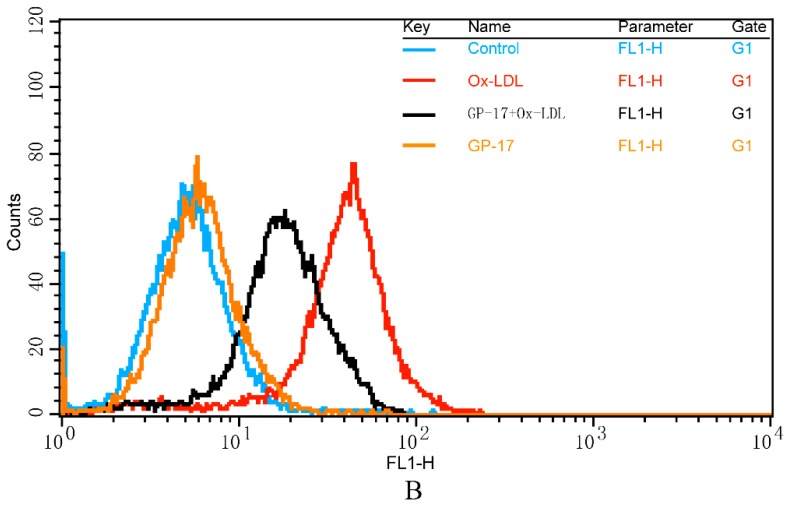
Effects of GP-17 on Ox-LDL-induced ROS production in HUVECs. (**A**) The ROS level was analyzed by fluorescence imaging; (**B**,**C**) Quantitative analysis of the fluorescence intensity of cells by flow cytometry. The values are expressed as the mean ± S.E.M. from three independent experiments. ^##^ *p* < 0.01 vs. Control; ** *p* < 0.01 vs. Ox-LDL.

Ox-LDL-induced ROS generation in HUVECs activates apoptotic signaling. Therefore, we investigated the effects of GP-17 on the generation of ROS in HUVECs using H2DCFDA as a fluorescence probe. In the presence of 100 μg/mL Ox-LDL, ROS generation markedly increased compared with that of the control cells. However, incubation of HUVECs with GP-17 for 12 h before exposure to Ox-LDL markedly reduced the ROS generation (Figure 7A,B).

### 2.6. Effect of GP-17 Treatment on Expression of ERα

**Figure 8:**
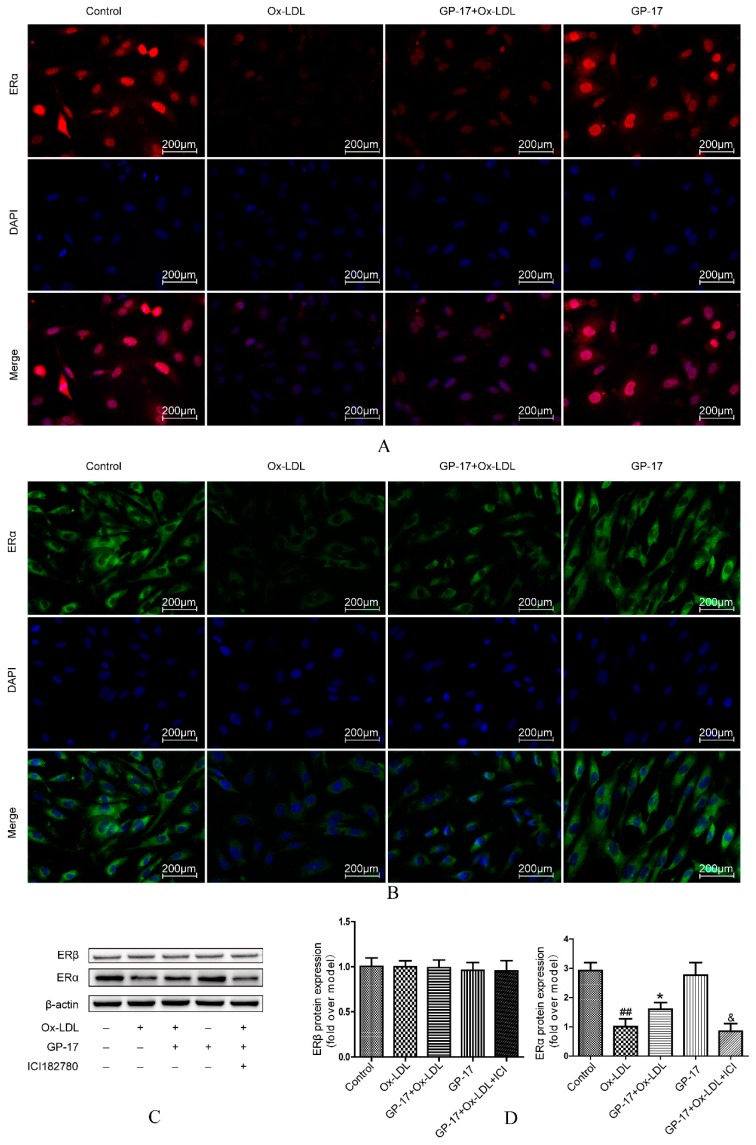
Effect of GP-17 on ERα in Ox-LDL-treated HUVECs. (**A**) Localization of ERα immunoreactivity was identified in the cytoplasm by immunofluorescence assays; (**B**) Localization of ERα immunoreactivity was identified in the plasma membrane by immunofluorescence assay; (**C**,**D**) The expressions of both ERα and ERβ were analyzed by western blots of Ox-LDL-induced HUVECs. The values are expressed as the mean ± S.E.M. from three independent experiments. ^##^ *p* < 0.01 vs. Control; * *p* < 0.05 vs. Ox-LDL; ^&^ *p* < 0.05 vs. GP-17 + Ox-LD. DAPI, 4,6-diamidino-2-phenylindole.

Our previous research found that GP-17 could activate estrogen receptors in PC12 cells, but there are no relevant reports in HUVECs. Therefore, we evaluated the expression of ERα and ERβ by western blot analysis. The results showed that ERα was significantly decreased in Ox-LDL-treated HUVECs compared with that of the control group, while incubation with GP-17 (50 μg/mL) substantially increased the expression of ERα. Compared with the control group, GP-17 alone did not significantly increase the expression of ERα. However, the expression of ERβ was scarcely affected in the different groups (Figure 8C,D). The data indicated that ERα was the predominant isoform in Ox-LDL-induced endothelial cell injury. To verify the increased ERα expression induced by GP-17, we pretreated HUVECs with the unspecific ER antagonist ICI182780 (10 nM). The inhibitor significantly abolished the protective effects (Figure 8C,D). In addition, the results of immunocytochemical analyses showed that GP-17 pretreatment increased ERα expression in both the plasma membrane and cytoplasm, and the expression of ERα was consistent with the results of western blot analysis (Figure 8A,B).

### 2.7. PI3K/Akt Activation by GP-17 via the ERα-Dependent Pathway

**Figure 9:**
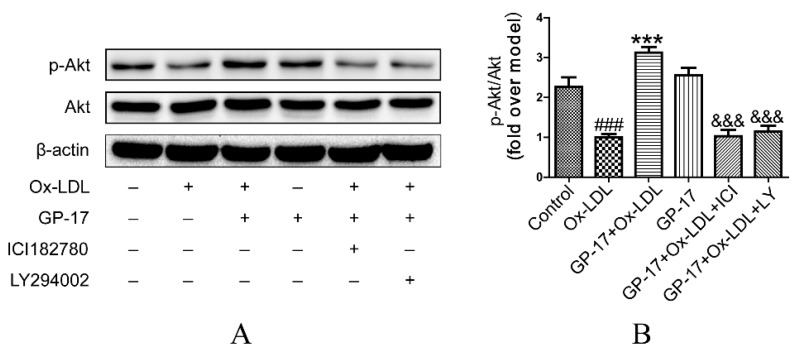
Effect of GP-17 on PI3K/Akt phosphorylation via ERα in Ox-LDL treated HUVECs. (**A**,**B**) Phosphorylation of Akt mediated by GP-17 in Ox-LDL-induced HUVECs was abolished by pretreatment with ICI182780 or LY294002. The values are expressed as the mean ± S.E.M. from three independent experiments. ^#^ *p* < 0.05; ^###^ *p* < 0.001 vs. Control; * *p* < 0.05; *** *p* < 0.001 vs. Ox-LDL; ^&^ *p* < 0.05; ^&&&^ *p* < 0.001 vs. GP-17 + Ox-LDL. p-Akt, phospho-protein kinase B.

To further explore the mechanism of GP-17 protection of HUVECs against Ox-LDL-induced injury, we examined the effect of GP-17 on the downstream signaling pathway of ERα. Regulation of the PI3K/Akt pathway by estrogen was reported to be mediated via estrogen receptors in HUVECs. Therefore, we evaluated the activation of Akt in GP-17-treated HUVECs by western blotting using specific antibodies. The expression of total Akt was not affected, as shown when similar amounts of proteins were loaded in each lane (Figure 9A,B). Compared with the control, phosphorylation of Akt (S473) markedly decreased in response to Ox-LDL-treated endothelial cells. However, pretreatment with GP-17 significantly increased phosphorylation of Akt compared with that of the Ox-LDL group. To address the role of the ERα in PI3K/Akt activation, we examined the effects of ICI182780 (10 nM) and LY294002 (12.5 nM) on the ERα/PI3K/Akt pathways, as inhibitors of the ERs or PI3K significantly block GP-17-induced Akt phosphorylation. These results suggested that GP-17 protected against Ox-LDL-induced endothelial cell injury through the ERα-mediated PI3K/Akt pathway.

### 2.8. GP-17 Increases Antioxidant Defense via the ERα/PI3K/Akt Pathway in HUVECs

**Figure 10:**
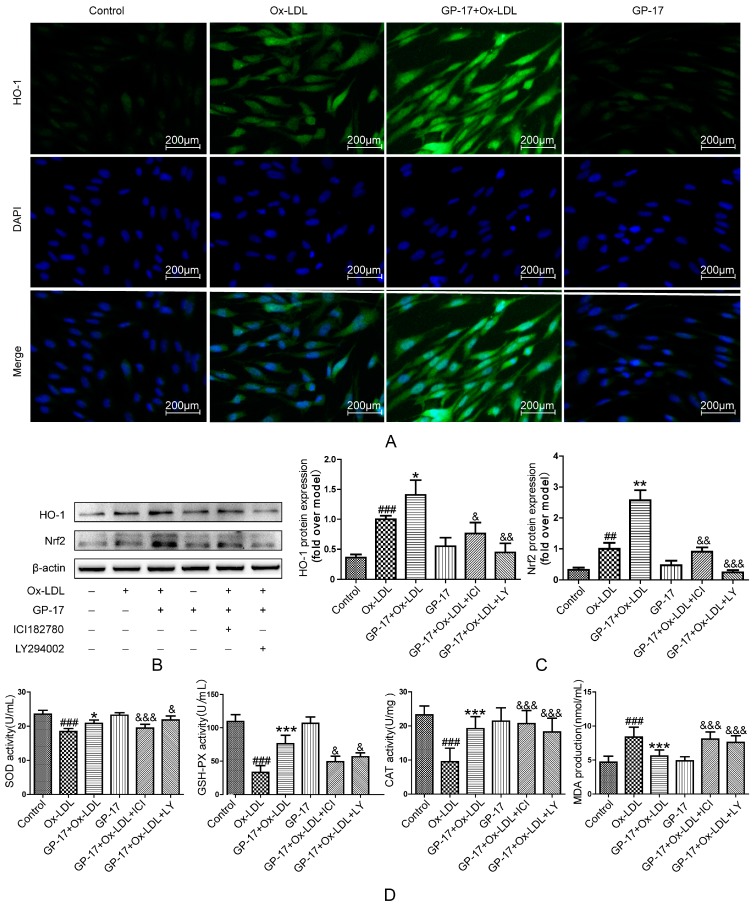
GP-17 protected HUVECs against Ox-LDL-induced oxidative stress by increasing the antioxidant enzymes and antioxidant proteins. (**A**) The expression of HO-1 was analyzed by immunofluorescence assays; (**B**,**C**) The expressions of antioxidant proteins mediated by GP-17 in Ox-LDL-induced HUVECs were abolished by pretreatment with ICI182780 or LY294002; (**D**) the levels of SOD, GSH-Px and CAT activities and MDA production mediated by GP-17 in Ox-LDL-induced HUVEC injury were determined and abolished by pretreatment with ICI182780 or LY294002. The values are expressed as the mean ± S.E.M. from three independent experiments. ^##^ *p* < 0.01; ^###^ *p* < 0.001 vs. Control; * *p* < 0.05; ** *p* < 0.01; *** *p* < 0.001 vs. Ox-LDL; ^&^ *p* < 0.05; ^&&^ *p* < 0.01; ^&&&^ *p* < 0.001 vs. GP-17 + Ox-LDL. HO-1, heme oxygenase 1; SOD, superoxide dismutase; GSH-Px, glutathione peroxidase; CAT, catalase; MDA, malondialdehyde.

Previous results showed that GP-17 could inhibit ROS production. To determine the mechanisms underlying GP-17’s antioxidative activity, we next assessed the expression of antioxidant enzymes in HUVECs. The results showed that the activities of SOD, GSH-Px and CAT significantly decreased when the HUVECs were exposed to Ox-LDL, whereas MDA markedly increased. However, pretreatment with GP-17 resulted in a substantial reversal of the SOD, GSH-Px and CAT activities and a significant reduction in the MDA production. Additionally, the antioxidant effect of GP-17 was eliminated when the HUVECs were pretreated with ICI182780 and LY294002 (Figure 10D). The data indicated that GP-17 partially regulates the oxidant sensitivity via the ERα/PI3K/Akt pathway.

In addition, HO-1 is a very important antioxidant protein and is controlled by Nrf2. In our previous work, we showed that GP-17 was involved in regulating the phosphorylation of Nrf2 and ARE-mediated phase II gene expression by activating the ERα/PI3K/Akt pathway in PC12 cells; thus, we examined the effect of GP-17 on Nrf2 activation and HO-1 protein expression. The results showed that the expressions of HO-1 and Nrf2 significantly increased in Ox-LDL-treated HUVECs compared with that of the control group. However, pretreatment with GP-17 resulted in increased expressions, but the Nrf2 and HO-1 protein expression was abolished by ICI182780 and LY294002, respectively (Figure 10B,C). We also found that there was scarcely any expression of these proteins in the control group and the GP-17 group. Furthermore, the results of immunocytochemical analyses showed that GP-17 pretreatment increased HO-1 expression, and these findings are consistent with those of western blot analyses (Figure 10A). These data indicated that GP-17 modulated HO-1 production and Nrf2 activation through the ERα-mediated PI3K/Akt pathway.

### 2.9. GP-17 Inhibits Ox-LDL-Induced HUVEC Apoptosis by Increasing Bcl-2 Family Proteins and Decreasing Caspase-3 Activation

**Figure 11:**
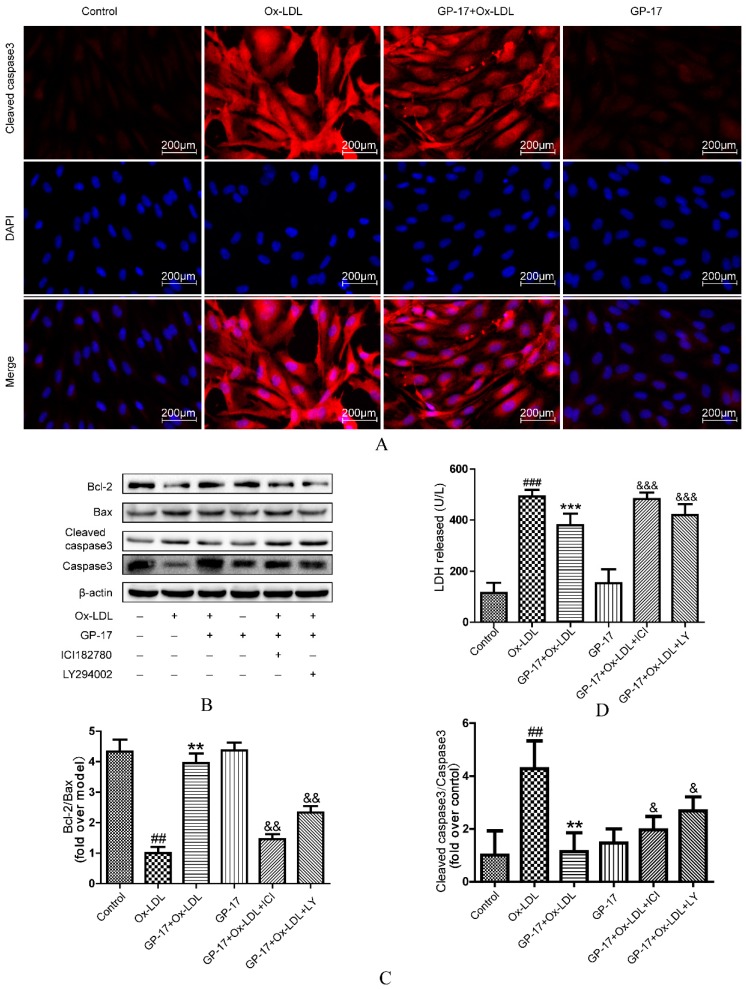
GP-17 protected HUVECs against Ox-LDL-induced apoptosis by decreasing the ratio of Bax to Bcl-2 and controlling cleaved caspase-3 activation. (**A**) The expression of cleaved caspase-3 was analyzed by immunofluorescence assays; (**B**,**C**) The expression of apoptosis-inducing proteins mediated by GP-17 in Ox-LDL-induced HUVECs was abolished by pretreatment with ICI182780 or LY294002; (**D**) The protective effect of GP-17 against LDH release induced by Ox-LDL in HUVECs. The values are expressed as the mean ± S.E.M. from three independent experiments. ^##^ *p* < 0.01; ^###^ *p* < 0.001 vs. Control; ** *p* < 0.01; *** *p* < 0.001 vs. Ox-LDL; ^&^ *p* < 0.05; ^&&^ *p* < 0.01; ^&&&^ *p* < 0.001 vs. GP-17 + Ox-LDL.

We next determined the mechanisms of GP-17’s anti-apoptosis effect. We found that Ox-LDL significantly decreased the ratio of Bcl-2 to Bax, while pretreatment with GP-17 reversed the down-regulation of Bcl-2 and up-regulation of Bax in HUVECs exposed to Ox-LDL (Figure 11B,C).

Moreover, cleaved caspase-3 is an executor of apoptosis in HUVECs in response to Ox-LDL. The results showed that Ox-LDL significantly increased cleaved caspase-3 in HUVECs, but this was suppressed by incubation with GP-17 (Figure 11B,C), and the expression of cleaved caspase-3 was very accordant to Caspase-3 Activity Assay (Figure S2). Furthermore, the results of immunocytochemical analyses also showed that GP-17 pretreatment decreased cleaved caspase-3 expression, and the result is consistent with the results of western blot analyses (Figure 11A). However, blockade of ERα and PI3K reversed the protective effect of GP-17. Additionally, LDH release, an indicator of endothelium injury, was evaluated in HUVECs (Figure 11D). In the Ox-LDL-treated group, LDH release was dramatically increased compared with that of the control group, whereas GP-17 treatment markedly inhibited it. These results indicated that GP-17 protected endothelial cells against Ox-LDL-induced apoptosis through by the ERα-mediated PI3K/Akt pathway.

## 3. Discussion

In the present study, we demonstrated for the first time that the antioxidant and anti-atherosclerotic effects of GP-17 in ApoE−/− mice were mediated by its phytoestrogen activity. Furthermore, our mechanistic studies showed that GP-17 inhibited Ox-LDL-induced HUVEC damage by increasing anti-apoptotic proteins and antioxidant protein expression through the ERα-mediated PI3K/Akt pathway. These results indicated that GP-17 could be a selective estrogen receptor alpha modulator for atherosclerosis treatment.

Gypenoside XVII (GP-17), a novel phytoestrogen belonging to the gypenosides, has a similar structure to that of estradiol. Prior studies have demonstrated that phytoestrogens could decrease the development and the progression of atherosclerotic lesions by improving the lipid profile and inhibiting oxidative stress and apoptosis [24,25,26]. Here, the effects of GP-17 on serum lipids were first studied using bioassays. We found that the estrogen-like activity of GP-17 could significantly lower serum levels of TC and LDL-C compared to those of the ApoE−/− control mice, but there was no effect on serum levels of TG and HDL-C. Our results were consistent with another study that showed that estrogen treatment significantly reduced serum levels of TC and LDL-C without changing TG and HDL-C [27]. Furthermore, GP-17 treatment substantially decreased lipid deposition and effectively stopped atherosclerosis formation in the ApoE−/− mice. The results indicated that lowering TC and LDL-C levels could prevent the progression of atherosclerosis. Nevertheless, GP-17 treatment did not have a significant effect on body weight. Additionally, we found that GP-17 exhibited protective effects against oxidative damage by reducing MDA content and increasing antioxidant enzymes, such as SOD, GSH-Px and CAT, in the blood of ApoE−/− mice. The data were consistent with our previous results demonstrating the antioxidative properties of GP-17 [16].

The physiological function of estrogen is mediated through its specific receptors, ERα, ERβ and GPER, and the activation of estrogen receptors can exert distinct responses via genomic signaling and nongenomic signaling as well as combined pathways depending on the specific subtype and subcellular distribution [13]. Previous studies showed that HUVECs expressed both ERα and ERβ [28]. However, in our study, GP-17 treatment predominantly regulated the expression of ERα but not ERβ. Localization of ERα immunoreactivity showed that there was a strong fluorescence signal in both the cell membrane and intracellularly, and these results were consistent with many studies showing that ERα is the predominant isoform [29]. What’s more, according to the results of MTT assay, the viability of HUVCEs pretreated with GP-17 at high concentrations (100 µg/mL) was significantly lower than 50 µg/mL groups after treated with Ox-LDL, in further study we found that this phenomenon is closely related to the expression of ERα. Our study found ERα expression peaked at 50 µg/mL GP-17 in HUVCEs and decreased gradually afterwards (Figure S3). Furthermore, we assessed whether GP-17 could activate the specific signaling pathways downstream of ERα. In our research, we found that GP-17 pretreatment significantly increased the expression of phosphorylation of PI3K and Akt, but the phosphorylation of Akt by GP-17 was blocked by the PI3K inhibitor and ERα inhibitor. The results indicated that GP-17 activated PI3K/Akt signaling pathway via an ERα-dependent mechanism in the Ox-LDL-induced HUVEC damage, which was consistent with other reports [30,31].

In the atheromatous process, overproduction of ROS has been regarded as the major cellular response to AS [32]. ROS production can activate lipid peroxidation and aggravate the formation of Ox-LDL [33]. Ox-LDL can further initiate the atherosclerotic process by induction of gene expression in HUVECs, which may result in apoptosis and activation of oxidative stress [34]. Therefore, antioxidant treatment is considered to be a plausible strategy to prevent atherosclerotic diseases. One of the most effective ways to suppress oxidative stress is to target freely scavenging superoxide anions and hydrogen peroxide by increasing the expression of antioxidant proteins and antioxidant enzymes. Previous studies have shown that ER-mediated PI3K/Akt activation stimulated Nrf2/ARE-regulated antioxidant protein expression and increased the expression of antioxidant enzymes [16,30,35]. In this study, our results showed that pretreatment with GP-17 resulted in a considerable increase in the SOD, GSH-Px and CAT activities and a significant reduction in the MDA production, but ICI182780 and LY294002 could reverse the protective effect of GP-17 against Ox-LDL-induced HUVEC damage. The data showed that GP-17 augmented antioxidant activity by the ERα/PI3K/Akt pathway in the Ox-LDL-induced HUVEC injury.

Endothelial cell apoptosis is another important characteristic of atherosclerosis. A previous study elucidated the mechanism of apoptosis in endothelial cells [6]. In this study, our findings showed that GP-17 inhibited Ox-LDL-mediated HUVEC apoptosis by reducing LDH release, restoring mitochondrial membrane potential, decreasing the ratio of Bax to Bcl-2 and controlling cleaved caspase-3 activation. However, this action of GP-17 could be blocked by ICI182780 and LY294002, and this could explain why GP-17 supplementation was able to inhibit apoptosis by the ERα/PI3K/Akt pathway in the Ox-LDL-induced HUVEC injury, which was consistent with previous reports [16,35]. However, the caspase-3 activities were significantly higher in LY294002 group than in ICI182780 group, but the situation was reversed in the consequence of LDH release. That is because the activation of caspase-3 represents an essential step in the apoptotic process, LY294002 is a inhibitor for PI3K, and ICI182780 is ERs antagonist. Although ERs is pathways upstream of PI3K [14], the inhibition effect of ICI182780 is worse than LY294002. That’s because GP-17 had other protection mechanism, so the inhibition of ERs downstream targets could result in more effective inhibition against GP-17 protection of HUVECs [36]. What’s more, LDH release is a consequence of damage to cellular integrity during apoptosis, which can indirectly reflect cytotoxicity. The data in my study indicated that the cytotoxicity of 10 nM ICI182780 was stronger than 12.5 nM LY294002, and the result was consistent with 3-(4,5-dimethylthiazol-2yl)-2,5-diphenyltetrazolium bromide (MTT) analysis (Figure S4).

However, this study also has disadvantages, for example, the molecular mechanism of GP-17 on serum lipids needs to be elucidated, and the antioxidant effect of GP-17 requires further examination in vivo.

## 4. Materials and Methods

### 4.1. Reagent and Materials

GP-17 (molecular weight = 947.158; purity > 98%) was from Shanghai Winherb Medical S&T Development (Shanghai, China). Six-week-old male ApoE−/− mice with a C57BL/6J background and C57BL/6J mice were purchased from the Experimental Animal Center of Beijing University of Medical Sciences (Beijing, China). Dimethyl sulfoxide (DMSO), oil red O and Collagenase I were obtained from Sigma-Aldrich (St. Louis, MO, USA). Total cholesterol (TC), triglyceride (TG), low-density lipoprotein cholesterol (LDL-C) and high-density lipoprotein cholesterol (HDL-C) commercial kits were obtained from Zhongsheng Bio-tech Co., Ltd. (Beijing, China). All of the cell culture materials were supplied from Lifeline (Frederick, MD, USA). Ox-LDL was obtained from Union-BioTechnology (Beijing, China), and [3-(4,5-dimethylthiazol-2yl)-2,5-diphenyltetrazolium bromide] (MTT) and fluorescent dye (JC-1) were obtained from Enzo Life Sciences (Plymouth Meeting, PA, USA). The AnnexinV/Propidium Iodide (PI) assay kit was purchased from Invitrogen (Eugene, OR, USA). The Image-iT^TM^ LIVE Green Reactive Oxygen Species Detection Kit and 4,6-diamidino-2-phenylindole (DAPI) were acquired from Life Technologies (Carlsbad, CA, USA). The kits for determining lactate dehydrogenase (LDH), superoxide dismutase (SOD), malondialdehyde (MDA), glutathione peroxidase (GSH-Px), catalase (CAT), and the Coomassie Protein Assay Kit were obtained from the Nanjing Jiancheng Institute of Biological Engineering (Nanjing, China). The caspase-3/CPP32 Fluorometric Assay Kit (K105) purchased from BioVision, Inc. (Mountain View, CA, USA).Protease Inhibitor Cocktail, BCA Protein Assay Kit, and Enhanced Chemiluminescence Western Blot Detection Kits were supplied by CWbiotech (Beijing, China). Specific kinase inhibitors, such as LY294002 (CID: 3973), were obtained from Calbiochem (Santiago, CA, USA), and ICI182780 (CID: 104741) was from Sigma-Aldrich (St. Louis, MO, USA). Some of the antibodies were obtained from Santa Cruz Biotechnology (Santa Cruz, CA, USA), and others were purchased from Abcam (Cambridge, UK). Other chemicals were obtained from Sigma (St. Louis, MO, USA).

### 4.2. Experimental Animals and Drug Administration

A total of 42 ApoE−/− mice and 12 male C57BL/6J mice with the same age and body weight (approximately 20 g in body weight and 6 weeks old) were randomly divided into 4 groups (12 mice for each group) and orally administered the following once a: C57 control group (vehicle; 0.5% sodium carboxymethylcellulose, CMC-Na); ApoE−/− model group (vehicle; 0.5% CMC-Na); ApoE−/− + GP-17 group (GP-17, 50 mg/kg via i.g.); ApoE−/− + probucol group (probucol, 2 mg/kg via i.g.). Probucol is an antioxidant drug used as a positive control. After two weeks of acclimatization, all the mice were fed a high-fat diet including 0.3% cholesterol and 20% pork fat for 10 weeks. They were maintained in pathogen-free conditions at approximately 22 ± 1 °C on a 12 h light–dark cycle with free access to food and water. The body weights were determined every two weeks. After 10 weeks of the treatments, all animals were anesthetized with pentobarbital sodium and killed after being deprived of food overnight. Serum was immediately separated from blood samples by centrifugation at 3600 rpm for 15 min, and the tissue samples (heart and aorta) were rapidly removed and frozen in −8 °C. All animal protocols were approved by the Research Ethics Committee of the Chinese Academy of Medical Sciences and Peking Union Medical College, Beijing, China (SCXK 2014-0001).

### 4.3. Plasma Lipids and Oxidative Stress Analysis

At the end of the experiment, the plasma levels of TC, HDL-C, LDL-C and TG were detected using biochemical kits. The activities of CAT, SOD, and GSH-PX and MDA content were measured by commercially available kits according to the manufacturer’s instructions.

### 4.4. Oil Red Staining

For determination of the severity of the lesions, a method of en face was used [37]. Briefly, the aortic roots were sliced into 6 μm serial cryostat sections in the aortic valve. The resultant aortic sinus cryosections (6 μm) were evaluated based on oil red O staining using Image-Pro Plus (6.0, Media Cybernetics, Rockville, MD, USA) software. At least 5 cryosections sections for each animal in one treatment group were evaluated, and the lesion was calculated from 8 different mice.

### 4.5. Cell Preparation and Culture

HUVECs were isolated from fresh human umbilical veins using 0.1% collagenase I. After dissociation, the cells were collected and cultured in VascuLife^®^ Medium (CID: LL0003) supplemented with 100 U/mL penicillin and 100 μg/mL streptomycin. All cell cultures were kept in a humidified incubator at 37 °C with 5% CO_2_, and the media was refreshed every three days. Cells from passages 3–7 were used for the subsequent experiments.

### 4.6. Cell Viability Assay

For the establishment of an Ox-LDL-induced apoptosis model and measurement of GP-17’s protective effect, the HUVECs were seeded in 96-well plates at a density of 10^5^ cells per ml and grown for 24 h. Then, the HUVECs were pretreated with GP-17 (6.25, 12, 25, 50, 100 μg/mL) for 12 h in serum-free endothelial cell basal medium, followed by incubation with Ox-LDL (100 μg/mL, 24 h) which did not have GP-17. After 24 h, the treated HUVECs were incubated with 5 mg/mL MTT in fresh medium for an additional 4 h. Absorbance was measured at 570 nm using a plate reader (Infinite M1000, Tecan, Sunrise, Austria).

### 4.7. Cell Apoptosis Assay by Annexin V-FITC/ Propidium Iodide (PI)

Cell apoptosis was measured using the Annexin V-FITC Apoptosis Detection kit (Invitrogen, Eugene, OR, USA) according to the manufacturer’s protocol. Briefly, at the end of the experiment, the treated cells were harvested and washed with cold phosphate-buffered saline (PBS) three times. Subsequently, 10^6^ cells were resuspended in 200 µL binding buffer and then incubated with 10 µL Annexin-V-FITC in the dark for 15 min at 37 °C. Finally, 5 µL PI and 300 mL binding buffer were added immediately, followed by the flow cytometry (BD, San Jose, CA, USA) analyses. The experiments were repeated in triplicate.

### 4.8. Measurement of Mitochondrial Membrane Potential (ΔΨm) Disruption by Plate Reader and Fluorescence Microscopy

For analysis of the effects of GP-17 on ΔΨm, the change in ΔΨm was detected by JC-1 staining. HUVECs (10^5^ cells per well) were cultured in 24-well plates. After treatment, the cells were harvested and incubated with JC-1 (10 µM final concentration) at 37 °C in the dark for 30 min. The cells were immediately observed using fluorescence microscopy (EVOS^®^ FL Color, Life Technologies).

For further analysis of the fluorescence intensity, the treated cells were resuspended in fresh medium containing JC-1 (10 µM final concentration) and incubated at 37 °C in the dark for 30 min. The green JC-1 signal was measured at wavelengths of 485 nm for excitation and 535 nm for emission, the red signal at wavelengths of 540 nm for excitation and 590 nm for emission. The fluorescence intensity changes were analyzed using the automatic plate reader (Infinite M1000, Tecan).

### 4.9. Measurement of ROS Activity by Plate Reader and Fluorescence Microscopy

Changes in ROS were measured by staining the cells with 2,7-dichlorofluorescein-diacetate (DCFH-DA) prior to observation with fluorescence microscopy and flow cytometry analysis. In brief, after treatment, the cells were harvested by trypsinization and resuspended in phosphate-buffered saline (PBS) buffer. Subsequently, the cells were centrifuged for 5 min at 400× *g*, and the supernatant was discarded. Finally, the effect of GP-17 on intracellular ROS levels was determined using an Image-iT™ LIVE Green Reactive Oxygen Species Detection Kit according to the manufacturer’s instructions (Carlsbad, CA, USA). Then, part of the sample was used to examine the fluorescence by fluorescence microscopy (EVOS^®^ FL Color). The rest of the sample was quantitatively analyzed by a plate reader.

### 4.10. Measurement of Caspase-3 Activity, MDA, CAT, GSH-Px, SOD Activity, LDH Release in HUVCEs

The activities of caspase-3 activity, SOD, GSH-Px and CAT LDH release, and MDA content were measured using commercial kits following the manufacturer’s instructions.

### 4.11. Immunocytochemical Analysis of Estrogen Receptor, HO-1 and Caspase-3

HUVECs were equally distributed to 24-well plates and grown for 24 h. At the end of the series of treatments, single HUVECs were washed with PBS and fixed with 4% paraformaldehyde (Sigma, St. Louiscity, MO, USA) for 10 min at room temperature and washed again with PBS. Subsequently, they were permeabilized with PBS containing 0.1% Triton X-100 for 10 min, washed again with PBS, blocked with goat serum for 1 h, and then incubated overnight with rabbit anti-ERα (they were not permeabilized), anti-cleaved caspase-3, and mouse anti-HO-1 primary antibodies (1:100 dilution) at 4 °C. The treated cells were then washed 5 times with PBS and were incubated with Alexa Fluor 488-conjugated antibody or Alexa Fluor 590-conjugated antibody (1:200) (Invitrogen, Eugene, OR, USA) for 2 h at room temperature. For visualization of the nuclei, the cells were incubated in 300 nM DAPI for 5 min. The images were captured using a fluorescence microscope (EVOS^®^ FL Color, Life Technologies, Carlsbadcity, CA, USA).

### 4.12. Western Blot Analysis

Protein expression was analyzed by western blot analysis. HUVCEs were seeded in 75 m^2^ culture plates and allowed to grow to 80% confluence, and then, the HUVECs were pretreated with GP-17 (50 μg/mL) for 12 h in serum-free endothelial cell basal medium. Subsequently, they were exposed to Ox-LDL (100 μg/mL) in basal medium for 24 h, and total cellular protein was extracted, separated by SDS–PAGE and transblotted onto PVDF membranes. Next, they were permeabilized with PBS containing 0.5% skim milk 2 h. Finally, the membranes were treated using standard procedures with primary and secondary antibodies. Protein expression was observed using Image Lab software (Bio-Rad, Hercules, CA, USA). The results shown are representatives of at least three independent experiments. The primary antibodies used were as follows: β-actin (C-2): sc-8432; ERα (HC-20): sc-543; ERβ (1531): sc-53494; PI3K (H-300): sc-67306; p-PI3K (Tyr 467): sc-293115. p-Akt1/2/3 (Ser 473): sc-101629; Akt1/2/3 (H-136): sc-8312; Nrf2: ab62352; HO-1: ab13248; Bcl-2 (N-19): sc-492; Bax (N-20): sc-493; caspase-9 p10 (H-83): sc-7885; caspase-3 p17 (S-19): sc-22139; cleaved caspase-3: ab2302.

### 4.13. Statistical Analysis

All the experiments were performed in triplicate. Data are expressed as the mean ± S.E.M. Statistical analysis was assessed using Student’s t *t*-test or ANOVA and conducted using SPSS (version 20.0, Chicago, IL, USA). *p* values less than 0.05 were considered significant.

## 5. Conclusions

**Figure 12:**
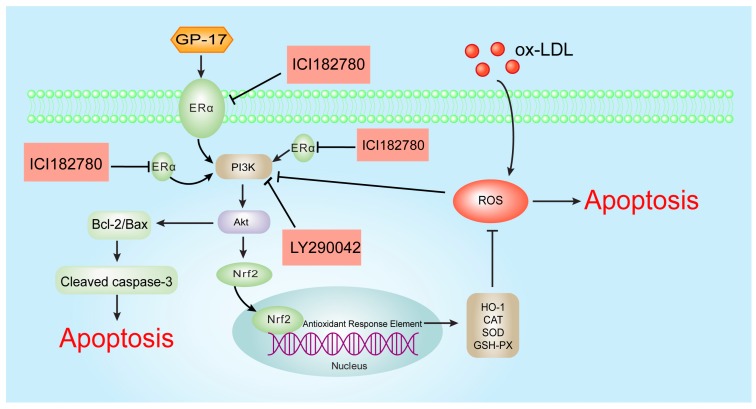
The molecular mechanism of the GP-17 anti-atherosclerotic effects. ROS induced by Ox-LDL result in endotheliocyte apoptosis and oxidative stress. GP-17 activates the ERα-mediated PI3K/Akt pathway, further resulting in Nrf2/HO-1 up-regulation and increasing the level of antioxidant enzymes. This process attenuates Ox-LDL-induced oxidative injury. Furthermore. GP-17 inhibited Ox-LDL-mediated HUVEC apoptosis by decreasing the ratio of Bax to Bcl-2 and controlling cleaved caspase-3 activation. This process inhibits Ox-LDL-induced endothelial cell apoptosis via the ERα-mediated PI3K/Akt pathway. T bars represented inhibition and arrows represented activation.

In conclusion, our study demonstrated that GP-17 could reduce the serum lipid levels, increase the expression of antioxidant enzymes and decrease atherosclerotic lesion formation in ApoE−/− mice. Furthermore, our mechanistic studies showed that GP-17 protected HUVECs against Ox-LDL-induced apoptosis and oxidative stress and that these effects are likely mediated by up-regulating the ERα-mediated PI3K/Akt pathway (Figure 12). Our findings indicate that GP-17 may be a new candidate agent for the prevention of atherosclerosis.

## Supplementary Materials

Supplementary materials can be found at www.mdpi.com/1422-0067/18/2/77/s1.

## Abbreviations

LY	LY294002
ICI	ICI182780
TG	Triglyceride
AS	Atherosclerosis
TC	Total cholesterol
CAT	Catalase
GP-17	Gypenoside XVII
ERα	Estrogen receptor alpha
ERβ	Estrogen receptor beta
GPER	G protein-coupled estrogen receptor 1
ECDs	Estrogen-dendrimer conjugates
ERs	Estrogen receptors
MDA	Malondialdehyde
HO-1	Heme oxygenase 1
ROS	Reactive oxygen species
SOD	Superoxide dismutase
PBS	Phosphate-buffered saline
LDH	Lactate dehydrogenase
GSH-Px	Glutathione peroxidase
PI3K	Phosphatidylinositide 3-kinase
Akt	Protein kinase B
DAPI	4,6-Diamidino-2-phenylindole
ApoE−/−	Apolipoprotein E-deficient
HUVECs	Human umbilical vein endothelial cells
Ox-LDL	Oxidized low-density lipoprotein
LDL-C	Low-density lipoprotein cholesterol
HDL-C	High-density lipoprotein cholesterol
Nrf2	Nuclear factor erythroid 2-related factor 2
MTT	3-(4,5-Dimethylthiazol-2yl)-2,5-diphenyltetrazolium bromide
JC-1	5,5’,6,6’-Tetrachloro-1,1’,3,3’-tetraethylbenzimidazolylcarbocyanine iodide
