# Supplementary material for: Gypenoside XVII Prevents Atherosclerosis by Attenuating Endothelial Apoptosis and Oxidative Stress: Insight into the ERα-Mediated PI3K/Akt Pathway
Source: Int J Mol Sci. 2017 Feb 9;18(2):77. doi: 10.3390/ijms18020077 (PMC5343768; doi:10.3390/ijms18020077)
Supplement: Supplementary file 1 [file ijms-18-00077-s001.pdf]

Supplementary Materials: Gypenoside XVII Prevents Atherosclerosis by Attenuating Endothelial Apoptosis and Oxidative Stress: Insight into the ERα-Mediated PI3K/Akt Pathway

Ke Yang, Haijing Zhang, Yun Luo, Jingyi Zhang, Min Wang, Ping Liao, Li Cao, Peng Guo, Guibo Sun and Xiaobo Sun

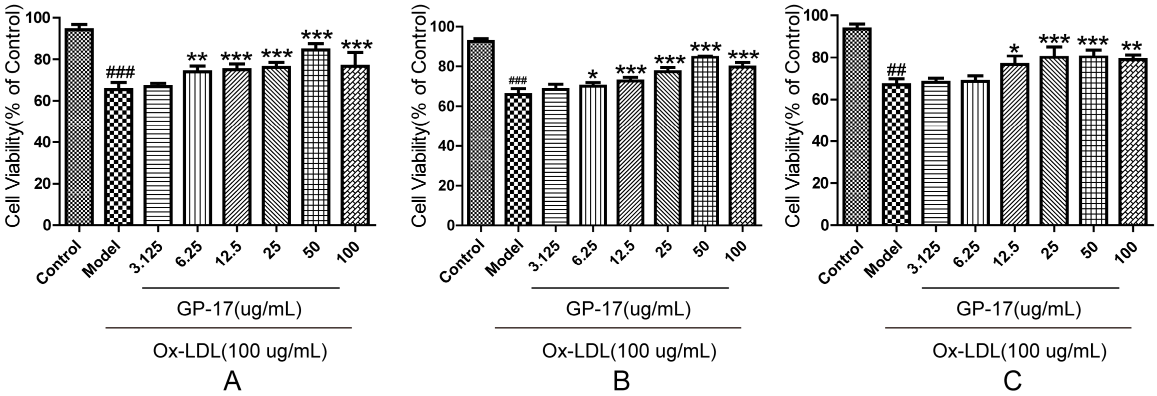

Figure S1. Cytoprotective effects for GP-17 on Ox-LDL-induced cytotoxicity in HUVECs. (**A**) Incubation with GP-17 for 4 h significantly lowered Ox-LDL-induced cell injury. Cell viability was measured by MTT assay; (**B**) Incubation with GP-17 for 8 h significantly lowered Ox-LDL-induced cell injury. Cell viability was measured by MTT assay; (**C**) Incubation with GP-17 for 24 h significantly lowered Ox-LDL-induced cell injury. Cell viability was measured by MTT assay. The values are expressed as the mean ± S.E.M. from three independent experiments. $^{\#\#}$ $p < 0.01$; $^{\#\#\#}$ $p < 0.001$ vs. Control; * $p < 0.01$; ** $p < 0.01$; *** $p < 0.001$ vs. Model.

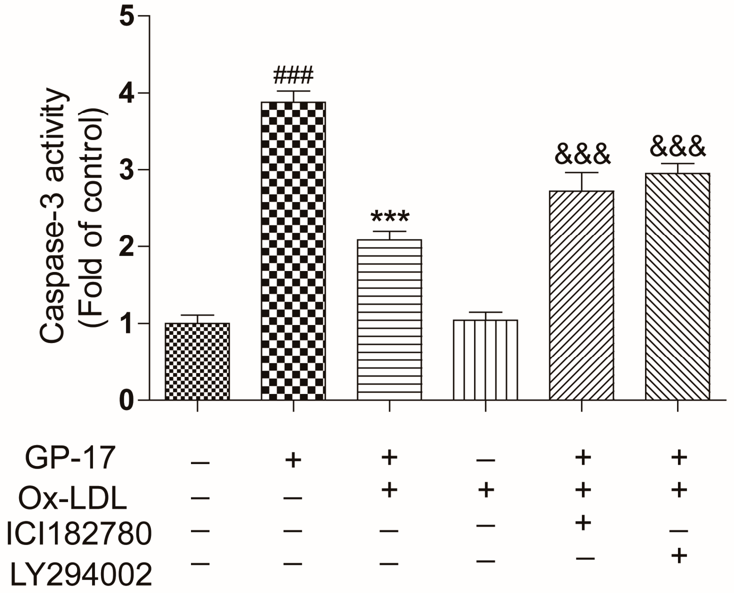

Figure S2. GP-17 protected HUVECs against Ox-LDL-induced apoptosis by decreasing caspase3 activity. Caspase3 activity mediated by GP-17 in Ox-LDL-induced HUVECs was abolished by pretreatment with ICI182780 or LY294002. $^{\#\#\#}$ $p < 0.001$ vs. Control; *** $p < 0.001$ vs. Ox-LDL; $^{\&\&\&}$ $p < 0.001$ vs. GP-17 + Ox-LDL.

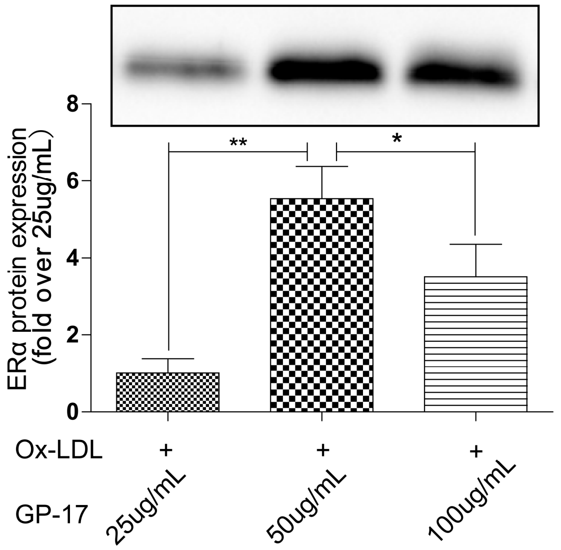

Figure S3. GP-17 had a dose-dependent on the expression of ERα in Ox-LDL treated HUVEC. ERα was mediated by pretreatment with different concentration of GP-17 in Ox-LDL-induced HUVECs. * $p < 0.05$; ** $p < 0.01$ vs. pretreatment with 50 μg/mL GP-17.

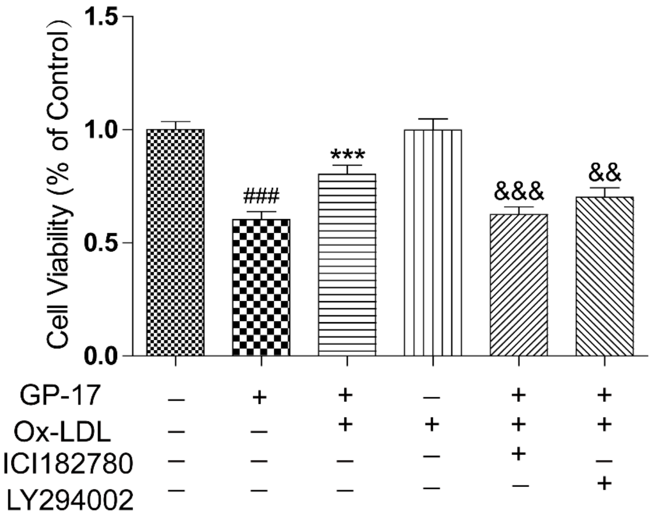

Figure S4. The activity of inhibitor analysis for ICI182780 and LY294002. Cytoprotective effect of GP-17 against Ox-LDL-induced cell viability reduction was inhibited by pretreatment with ICI-182,780 or LY294002. Cell viability was measured by MTT assay. ### $p < 0.001$ vs. Control; *** $p < 0.001$ vs. Ox-LDL; && $p < 0.01$; &&& $p < 0.001$ vs. GP-17 + Ox-LDL.
